# Emerging immunotherapies in osteosarcoma: from checkpoint blockade to cellular therapies

**DOI:** 10.3389/fimmu.2025.1579822

**Published:** 2025-03-18

**Authors:** Zhiwei Han, Guomin Chen, Dongchen Wang

**Affiliations:** ^1^ Department of Orthopedics, Central Hospital Affiliated to Shandong First Medical University, Jinan, China; ^2^ Laboratory Medicine, Central Hospital Affiliated to Shandong First Medical University, Jinan, China

**Keywords:** osteosarcoma, immunotherapy, checkpoint blockade, immune cell therapy, tumor microenvironment

## Abstract

Osteosarcoma remains a highly aggressive bone malignancy with limited therapeutic options, necessitating novel treatment strategies. Immunotherapy has emerged as a promising approach, yet its efficacy in osteosarcoma is hindered by an immunosuppressive tumor microenvironment and resistance mechanisms. This review explores recent advancements in checkpoint blockade, cellular therapies, and combination strategies aimed at enhancing immune responses. We highlight key challenges, including tumor heterogeneity, poor immune infiltration, and the need for predictive biomarkers. By integrating immunotherapy with chemotherapy, radiotherapy, and targeted therapy, emerging approaches seek to improve treatment outcomes. This review provides a comprehensive analysis of the evolving landscape of osteosarcoma immunotherapy, offering insights into future directions and potential breakthroughs. Researchers and clinicians will benefit from understanding these developments, as they pave the way for more effective and personalized therapeutic strategies in osteosarcoma.

## 1 Introduction

Osteosarcoma is the most common primary malignant bone tumor, predominantly affecting children, adolescents, and young adults (1). The global incidence of osteosarcoma is estimated at 3–5 cases per million annually, with a peak occurrence in the second decade of life, coinciding with periods of rapid bone growth. Despite significant advances in multimodal treatment strategies, including surgery and chemotherapy, the long-term prognosis for patients with metastatic or recurrent osteosarcoma remains poor (2). The current five-year survival rate for localized osteosarcoma approaches 60–70%, whereas it drops to less than 30% in cases with metastasis, particularly involving the lungs. The aggressive nature of osteosarcoma, coupled with its high propensity for distant dissemination and resistance to conventional therapies, underscores the urgent need for novel and more effective treatment approaches. Historically, the standard-of-care for osteosarcoma has comprised neoadjuvant chemotherapy, surgical resection, and adjuvant chemotherapy. While this regimen has improved survival rates over past decades, it has largely reached a therapeutic plateau, with minimal advances in overall survival observed in recent years. Moreover, the efficacy of traditional cytotoxic agents is often hindered by severe adverse effects, multidrug resistance, and inadequate control of micro-metastatic disease. Radiotherapy, though occasionally used in unresectable or palliative settings, has limited effectiveness due to the relative radioresistance of osteosarcoma cells (3). Given these limitations, there is a pressing demand for alternative therapeutic strategies that can enhance treatment efficacy while minimizing systemic toxicity.

Immunotherapy has emerged as a promising approach to overcoming the limitations of conventional treatments by leveraging the body’s immune system to recognize and eradicate tumor cells (4). Recent advances in tumor immunology have revealed that osteosarcoma, despite being historically considered immunologically “cold,” exhibits an immunosuppressive tumor microenvironment (TME) that can be modulated to improve immune responsiveness (5, 6). Among immunotherapeutic strategies, immune checkpoint blockade (ICB) targeting the programmed cell death protein 1 (PD-1)/PD-1 ligand (PD-L1) and cytotoxic T-lymphocyte antigen 4 (CTLA-4)pathways has demonstrated clinical success in several cancers and is now being actively explored in osteosarcoma (7, 8). Additionally, cellular therapies, including chimeric antigen receptor (CAR)-T cells, CAR-natural killer (NK) cells, and dendritic cell (DC) vaccines, offer novel avenues for enhancing anti-tumor immunity (9, 10). Given the complex interplay between immune evasion mechanisms in OS, combination strategies integrating immunotherapy with chemotherapy, radiotherapy, or targeted agents are being investigated to potentiate therapeutic efficacy.

This review provides a comprehensive overview of emerging immunotherapies in osteosarcoma, with a particular focus on checkpoint blockade, cellular therapies, and combinatorial approaches. By discussing current advancements, challenges, and future directions, we aim to highlight the potential of immunotherapy in reshaping the treatment landscape for OS and improving patient outcomes.

## 2 The immunosuppressive microenvironment of osteosarcoma

OS is characterized by a highly immunosuppressive TME, which plays a crucial role in tumor progression, immune evasion, and therapeutic resistance. Unlike immunologically “hot” tumors that are heavily infiltrated with cytotoxic T cells, osteosarcoma exhibits a predominantly immune-excluded or immune-suppressed phenotype, limiting the efficacy of immunotherapy (11, 12). The osteosarcoma TME is composed of various immune cells, including tumor-associated macrophages (TAMs), T cells, NK cells, and regulatory T cells (Tregs), which collectively contribute to an immunosuppressive niche. Additionally, osteosarcoma cells exploit immune checkpoint pathways, such as PD-1/PD-L1, CTLA-4, lymphocyte activation gene-3(LAG-3), and T cell immunoglobulin and mucin domain-containing protein 3(TIM-3), to evade immune surveillance. The cytokine and chemokine milieu further modulates immune cell infiltration and activity, reinforcing the immune-suppressive landscape of the tumor. Understanding the mechanisms underlying this immunosuppressive TME is critical for developing effective immunotherapeutic strategies for osteosarcoma.

### 2.1 Immune cell in the osteosarcoma tumor microenvironment

The cellular composition of the osteosarcoma TME plays a pivotal role in dictating the immune response. Various immune cell populations, including TAMs, T cells, NK cells, and Tregs, contribute to the immunosuppressive state of osteosarcoma, promoting tumor progression and resistance to immune-based therapies.

#### 2.1.1 Tumor-associated macrophages

TAMs are a dominant immune cell population in osteosarcoma, often displaying an M2-like, pro-tumorigenic phenotype. These macrophages promote tumor progression through multiple mechanisms, including immune suppression, angiogenesis, and extracellular matrix remodeling (13, 14). TAMs in osteosarcoma secrete high levels of immunosuppressive cytokines such as interleukin-10 (IL-10) and transforming growth factor-beta (TGF-β), which inhibit the activation and proliferation of cytotoxic T lymphocytes (CTLs) and NK cells (15). For instance, high infiltration of CD163^+^ M2-like macrophages in osteosarcoma correlates with poor prognosis and increased metastatic potential (16). Additionally, TAMs contribute to immune evasion by upregulating the expression of PD-L1, which interacts with PD-1 on T cells to induce T cell exhaustion (17). Targeting TAMs with colony-stimulating factor receptor (CSF1R) inhibitors or repolarizing them toward an M1-like phenotype using toll-like receptor (TLR) agonists has been proposed as a potential strategy to overcome macrophage-mediated immunosuppression in osteosarcoma (18).

#### 2.1.2 T cells: dysfunction and exhaustion in osteosarcoma

Although osteosarcoma tumors contain T cell infiltrates, their functionality is severely compromised. CD8^+^ cytotoxic T cells, which are critical for anti-tumor immunity, exhibit reduced effector function due to chronic antigen exposure and persistent immune checkpoint signaling. The phenomenon of T cell exhaustion is well-documented in osteosarcoma, with tumor-infiltrating T cells expressing high levels of PD-1, TIM-3, and LAG-3—markers associated with dysfunctional and anergic T cells (19, 20). A study analyzing osteosarcoma biopsies found that while CD8^+^ T cells were present in the TME, they lacked the ability to proliferate and secrete effector cytokines such as interferon-gamma (IFN-γ) and tumor necrosis factor-alpha (TNF-α) (19, 21). Moreover, the presence of CD4^+^ helper T cells is often skewed towards Treg phenotype, which further suppresses cytotoxic responses (22). Efforts to restore T cell function in osteosarcoma have focused on checkpoint blockade therapy, but the response rates have remained suboptimal, suggesting the need for combination strategies to reinvigorate T cell-mediated immunity.

#### 2.1.3 Natural killer cells: limited cytotoxicity in osteosarcoma

NK cells are innate immune effectors capable of recognizing and eliminating tumor cells without prior antigen sensitization (23). However, in osteosarcoma, the activity of NK cells is significantly suppressed due to multiple factors. Tumor-derived TGF-β downregulates the expression of activating receptors such as NKG2D on NK cells, impairing their ability to recognize and kill osteosarcoma cells (24). Furthermore, osteosarcoma cells frequently shed soluble ligands for NKG2D, further inhibiting NK cell-mediated cytotoxicity. Increasing NK cell infiltration through adoptive NK cell transfer or cytokine stimulation (e.g., IL-15 administration) can enhance anti-tumor immunity in osteosarcoma (25). Additionally, CAR-NK cell therapies targeting osteosarcoma-associated antigens such as B7-H3 are under investigation as potential strategies to overcome NK cell dysfunction in osteosarcoma (26).

#### 2.1.4 Regulatory T cells: a major suppressor of anti-tumor immunity

Tregs play a crucial role in maintaining immune tolerance, but in the context of cancer, they act as potent suppressors of anti-tumor immunity. osteosarcoma tumors often exhibit an increased frequency of CD4^+^CD25^+^FoxP3^+^ Tregs, which suppress the activity of CTLs and NK cells through the secretion of IL-10 and TGF-β (27, 28). Tregs interact with osteoblastic, endothelial, and myeloid cells through C-X-C motif chemokine ligand (CXCL) signaling, particularly affecting the expression of C-X-C motif chemokine receptor 4 (CXCR4). These interactions, primarily mediated by CXCL12 and transforming growth factor β1 (TGFB1), collectively facilitate tumor growth and progression (28). Additionally, Tregs express high levels of CTLA-4, which competes with CD28 for binding to B7 molecules on antigen-presenting cells, thereby dampening T cell activation. Agents such as anti-CD25 antibodies and CTLA-4 inhibitors are being explored for their potential to reduce Treg-mediated immunosuppression and restore effective anti-tumor responses.

### 2.2 Cytokine and chemokine landscape in osteosarcoma

The cytokine and chemokine landscape in osteosarcoma play a pivotal role in shaping immune cell recruitment and function, ultimately influencing tumor progression and immune evasion. Immunosuppressive cytokines such as TGF-β and IL-10 are prominently involved in promoting Treg expansion and suppressing effector T cell activity, thereby fostering an immunosuppressive TME. Conversely, pro-inflammatory cytokines like IFN-γ and IL-12 are critical for enhancing anti-tumor immunity; however, their expression is frequently suppressed in osteosarcoma, limiting their protective effects. Chemokines further contribute to this complex interplay: CCL2 and CCL5 facilitate the recruitment of TAMs and Tregs, while CXCL9 and CXCL10, which are essential for promoting T cell infiltration, are often downregulated in osteosarcoma (29, 30). To counteract these immunosuppressive mechanisms, therapeutic strategies aimed at modulating the cytokine and chemokine milieu are under investigation. For instance, IL-12 gene therapy has shown potential in restoring anti-tumor immunity (31), and CXCR4 antagonists are being explored to disrupt chemokine-mediated immune suppression (32). Osteosarcoma cells also secrete CXCL14 that activates integrinα11β1 on fibroblasts to form a lung metastatic niche (33).

## 3 Checkpoint blockade in osteosarcoma

Immune checkpoint blockade has revolutionized cancer immunotherapy by enhancing the immune system’s ability to recognize and eliminate tumor cells (34). However, in osteosarcoma, the efficacy of checkpoint inhibitors remains suboptimal due to the inherently immunosuppressive TME and various resistance mechanisms. Although osteosarcoma has historically been classified as an immunologically “cold” tumor with low levels of T cell infiltration, growing evidence suggests that modulating immune checkpoints reshape the TME and enhance anti-tumor immunity (7). The most widely studied immune checkpoints in osteosarcoma include PD-1/PD-L1 pathway and the CTLA-4 pathway. However, newer checkpoint molecules such as LAG-3, TIM-3, T cell immunoreceptor with Ig and ITIM domains (TIGIT), V-domain Ig suppressor of T cell activation (VISTA), and B7-H3 (CD276) have gained increasing attention as potential targets in osteosarcoma.

### 3.1 PD-1/PD-L1 inhibitors in osteosarcoma: clinical trials, efficacy, and limitations

The PD-1/PD-L1 axis plays a critical role in immune evasion in osteosarcoma. PD-L1 is frequently upregulated on osteosarcoma cells, particularly in response to IFN-γ signaling. Engagement of PD-L1 with PD-1 on T cells leads to T cell exhaustion, reducing their ability to proliferate and produce effector cytokines such as IFN-γ and TNF-α.

#### 3.1.1 Clinical trials and outcomes

Clinical trials evaluating PD-1 inhibitors (e.g., pembrolizumab, nivolumab) and PD-L1 inhibitors (e.g., atezolizumab, durvalumab) in osteosarcoma patients have demonstrated mixed outcomes. The SARC028 trial (NCT02301039), a phase II study investigating pembrolizumab in bone and soft tissue sarcomas, reported limited efficacy in osteosarcoma, with an overall response rate (ORR) of only 5% (35). Similarly, nivolumab monotherapy trials have shown minimal clinical benefit, as disease progression was observed in the majority of osteosarcoma patients (36). However, these trials have limitations, including small sample sizes, lack of biomarker-driven patient selection, and heterogeneous treatment responses. For example, most trials do not stratify patients based on PD-L1 expression levels, tumor mutational burden (TMB), or the composition of TME, which may impact treatment outcomes. In contrast, combination approaches have shown more promising results; for example, a trial combining pembrolizumab with axitinib, a VEGFR inhibitor, demonstrated improved disease control, suggesting that targeting the tumor vasculature may enhance the efficacy of immunotherapy in osteosarcoma (37). These findings highlight the potential of combination strategies to overcome the limitations of monotherapy in this challenging disease.

#### 3.1.2 Limitations of PD-1/PD-L1 blockade in osteosarcoma

The limited efficacy of PD-1/PD-L1 blockade in osteosarcoma can be attributed to several key factors. First, osteosarcoma exhibits a relatively low TMB compared to malignancies such as melanoma or lung cancer, resulting in fewer neoantigens available for immune recognition (38). Low TMB reduces the likelihood of generating sufficient tumor-specific neoantigens that can be recognized by T cells, thereby limiting the efficacy of immune checkpoint blockade. Recent studies suggest that enhancing TMB through genetic or epigenetic modifications, such as using DNA methyltransferase inhibitors, may improve response rates to checkpoint inhibitors in osteosarcoma. Second, deficiencies in antigen presentation further contribute to resistance. Osteosarcoma cells often downregulate major histocompatibility complex class I (MHC-I) molecules, which are critical for presenting tumor antigens to cytotoxic T cells. Loss of MHC-I expression impairs immune recognition, leading to immune evasion. Strategies to restore antigen presentation, such as IFN-γ treatment or histone deacetylase inhibitors (HDACis), have been explored as potential approaches to enhance immune checkpoint therapy efficacy in osteosarcoma. Third, the immunosuppressive TME in osteosarcoma inhibits T cell function and undermine anti-tumor immunity (6). Fourth, heterogeneous PD-L1 expression among osteosarcoma patients further limits the uniform effectiveness of PD-1/PD-L1 blockade, as not all patients exhibit sufficient PD-L1 levels to benefit from this therapy (39). To address these challenges, combination strategies that target multiple resistance mechanisms are actively being explored. For example, combining PD-1/PD-L1 blockade with therapies aimed at increasing antigen presentation (e.g., HDAC inhibitors), reprogramming the TME (e.g., CSF1R inhibitors, IL-10 blockade), or enhancing TMB through epigenetic modulation (e.g., DNA methyltransferase inhibitors) represents a promising approach to improve therapeutic outcomes.

### 3.2 CTLA-4 blockade: potential benefits and combination approaches

CTLA-4 is an inhibitory receptor expressed on T cells that competes with CD28 for binding to B7 molecules on antigen-presenting cells (APCs). By preventing co-stimulatory signaling, CTLA-4 inhibits T cell activation and expansion. Preclinical and clinical studies investigating CTLA-4 inhibitors in osteosarcoma have yielded limited but promising insights. Ipilimumab, an anti-CTLA-4 antibody, has demonstrated significant efficacy in melanoma; however, its clinical benefit in osteosarcoma remains uncertain. A pilot study evaluating ipilimumab in pediatric sarcomas reported modest disease stabilization, suggesting potential yet limited activity in osteosarcoma. Similarly, tremelimumab, another CTLA-4 inhibitor, has been tested in combination with PD-L1 inhibitors, but no significant survival benefit has been observed in osteosarcoma to date (40). These findings highlight the need for further research to optimize CTLA-4 blockade strategies, potentially through combination therapies or biomarker-driven patient selection, to enhance therapeutic outcomes in osteosarcoma.

### 3.3 Dual checkpoint inhibition: synergistic effects and challenges

#### 3.3.1 Dual targeting PD-1 and CTLA-4

Combination strategies are being explored to improve the limited efficacy of CTLA-4 monotherapy. One such approach is combining CTLA-4 blockade with PD-1 inhibition. In melanoma, the combination of ipilimumab (CTLA-4 inhibitor) and nivolumab (PD-1 inhibitor) has shown significant improvements in survival rates compared to monotherapy (41, 42). A phase I/II trial evaluated the combination of nivolumab and ipilimumab in children and young adults with recurrent/refractory osteosarcoma tumors. The study tested two dose levels (DL1 and DL2) for safety and established the recommended phase II dose (RP2D) for pediatric patients. However, this trial also faced challenges, including immune-related adverse events (irAEs), variability in response rates, and the need for longer follow-up to determine overall survival benefits. The RP2D combination was well tolerated and demonstrated some clinical activity in these pediatric patients with solid tumors (43). Preclinical studies in osteosarcoma have also suggested that dual checkpoint inhibition could enhance anti-tumor immunity. Another promising combination is CTLA-4 blockade with radiotherapy. Radiation therapy, by inducing immunogenic cell death, can increase tumor antigen release and immune cell infiltration. For example, studies using murine models have shown that the combination of CTLA-4 blockade and radiation therapy results in improved tumor control and enhanced immune responses, suggesting this combination may be effective in treating various cancers. Future trials are focusing on refining patient selection criteria by incorporating immune profiling and genomic analyses to identify responders more accurately. For example, assessing tumor neoantigen load and immune cell infiltration levels may help predict which patients will benefit most from dual checkpoint blockade.

#### 3.3.2 Targeting LAG-3 and PD-1 in osteosarcoma

LAG-3 is co-expressed with PD-1 on exhausted T cells and contributes to immune suppression, making it an attractive target for immune checkpoint therapy (44, 45). Blocking LAG-3 enhances T cell proliferation and cytokine production, suggesting a potential strategy to restore immune function in tumors (46). LAG-3 is highly expressed in osteosarcoma (47).One promising combination is the use of relatlimab (anti-LAG-3) alongside nivolumab (anti-PD-1), which has shown encouraging results in melanoma and other solid tumors (48, 49). This combination is now being investigated in osteosarcoma to determine its potential efficacy (50). Preclinical models of osteosarcoma have demonstrated that LAG-3 blockade, when combined with PD-1 inhibition, results in increased tumor regression in murine models, further supporting the therapeutic potential of targeting LAG-3 in osteosarcoma.

#### 3.3.2 TIM-3 Blockade in osteosarcoma

TIM-3 is another key exhaustion marker on T cells and plays a significant role in immune evasion (51). It is also expressed on TAMs, where it contributes to their immunosuppressive function (52). A phase I/II study evaluated the safety and efficacy of sabatolimab (MBG453), an anti-TIM-3 monoclonal antibody, with or without spartalizumab (anti-PD-1), in patients with advanced solid tumors. The results showed that sabatolimab plus spartalizumab was well tolerated and demonstrated preliminary signs of antitumor activity (53). But studies in osteosarcoma for co-blockade of TIM-3 and PD-1 require further study. It could be a promising strategy to overcome immune suppression and improve therapeutic outcomes in osteosarcoma.

#### 3.3.3 Targeting TIGIT in osteosarcoma

TIGIT is an emerging immune checkpoint that suppresses the activity of both NK cells and T cells, contributing to immune evasion in tumors (54). Tiragolumab, an anti-TIGIT antibody, has shown promise when combined with PD-L1 blockade in non-small cell lung cancer and is currently being investigated in osteosarcoma (55). In preclinical models of osteosarcoma, blocking TIGIT has been shown to enhance NK cell-mediated cytotoxicity, suggesting that targeting TIGIT could improve the anti-tumor immune response (56, 57). This combination strategy holds potential for overcoming immune suppression and improving treatment outcomes in osteosarcoma.

### 3.4 Mechanisms of resistance and strategies to enhance response rates

Despite the promise of checkpoint inhibitors, resistance remains a significant challenge in cancer therapy. Several mechanisms of resistance have been identified. The low tumor mutational burden (TMB) of osteosarcoma leads to reduced neoantigen availability, limiting T cell recognition and immune activation. Enhancing TMB through mutagenic therapies or epigenetic modulation has been proposed to overcome this challenge. Additionally, antigen presentation deficiencies, particularly the downregulation of MHC-I molecules, impair T cell recognition of osteosarcoma cells. Strategies such as IFN-γ stimulation or the use of histone deacetylase inhibitors (HDACis) have been explored to restore MHC-I expression and improve antigen presentation. The adaptive upregulation of other immune checkpoints, such as LAG-3, TIM-3, or VISTA, following PD-1 blockade, necessitates the development of multi-target approaches to overcome this compensatory immune evasion. Additionally, TME-mediated resistance, characterized by the suppression of T cell activation through MDSCs, Tregs, and TAMs, further contributes to immune escape. Additionally, deficient antigen presentation, such as the downregulation of MHC-I molecules in osteosarcoma cells, impairs T cell recognition and limits the effectiveness of immune checkpoint inhibitors. Addressing these resistance mechanisms through combination therapies and strategies to enhance antigen presentation is critical for improving response rates and overcoming therapeutic resistance.

Several strategies have been proposed to overcome resistance to checkpoint inhibitors. Epigenetic modulators, such as hypomethylating agents, upregulate immune-related genes, thereby enhancing the efficacy of checkpoint inhibitors. Cytokine-based therapies, including IL-12 and IL-15, promote the activation of T cells and NK cells, potentially improving immune responses (58). Additionally, oncolytic viruses, which are engineered to selectively infect and kill tumor cells, can increase tumor antigen release, further enhancing the efficacy of immune checkpoint blockade.

## 4 Immune cell therapies for osteosarcoma

### 4.1 CAR-T cell therapy

CAR-T cell therapy has demonstrated remarkable success in hematologic malignancies, yet its efficacy in solid tumors like osteosarcoma remains limited (59, 60). The development of CAR-T therapy for osteosarcoma has focused on identifying suitable tumor-associated antigens (TAAs) that are highly expressed in osteosarcoma cells while sparing normal tissues. However, despite promising preclinical data, clinical translation has been challenging due to factors such as antigen heterogeneity, T cell exhaustion, and the immunosuppressive tumor.

#### 4.1.1 Target antigens in osteosarcoma

**Figure 1:**
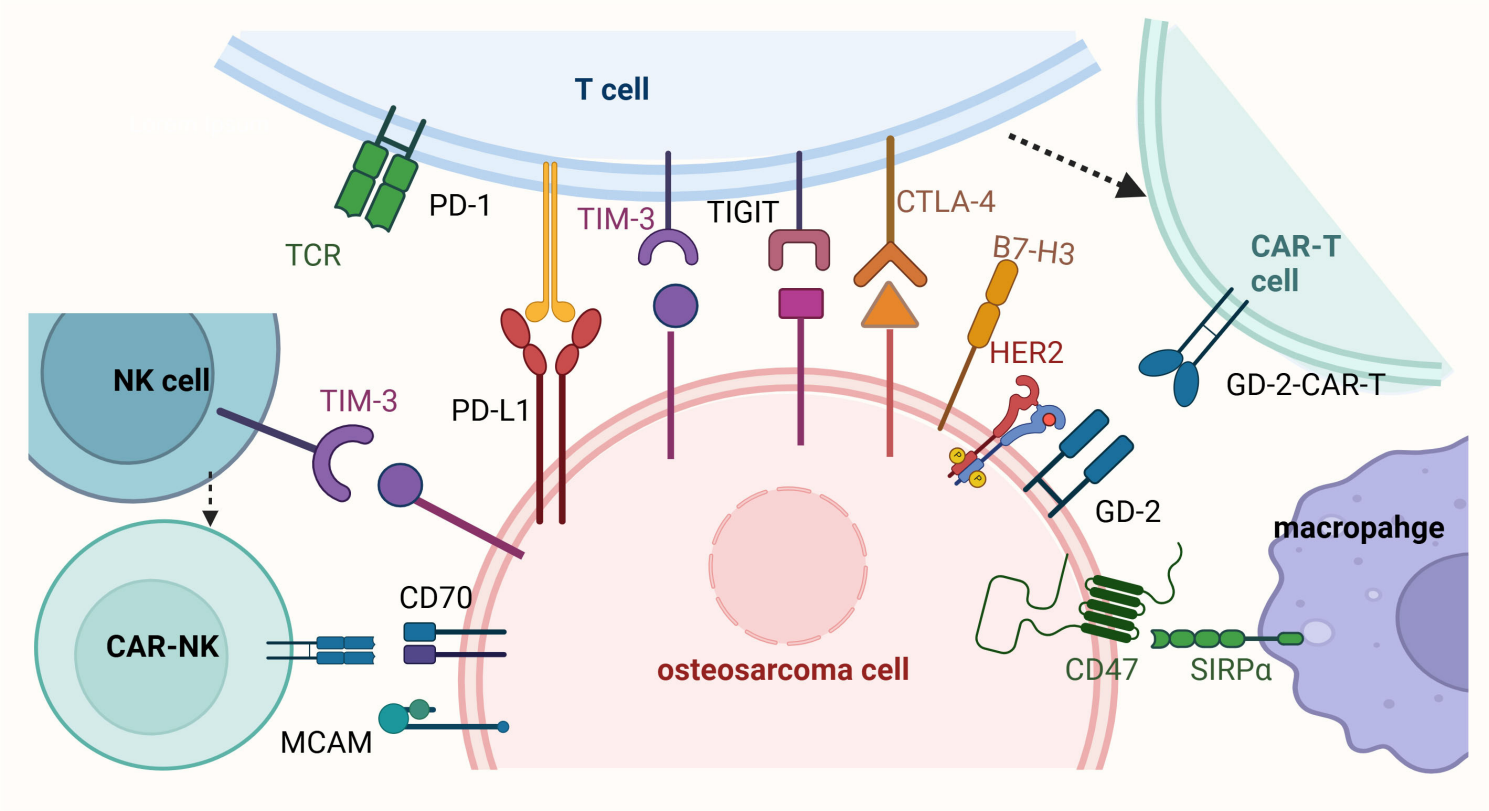
Immunotherapy and cell therapy for osteosarcoma. This figure illustrates the mechanisms of immune evasion in osteosarcoma and highlights potential immunotherapeutic and cell-based therapeutic strategies. T cells express immune checkpoint receptors such as PD-1, CTLA-4, TIM-3, and TIGIT, which interact with their respective ligands (e.g., PD-L1 and B7-H3) on osteosarcoma cells, leading to T cell exhaustion and immune evasion. The presence of HER2-CAR-T and GD2-CAR-T cells represents promising adoptive cell therapy approaches to enhance T cell-mediated tumor killing. Macrophages interact with osteosarcoma cells through the CD47-SIRPα axis, a “don’t eat me” signal that inhibits phagocytosis; blocking this pathway is a potential strategy to enhance macrophage-mediated tumor clearance. Osteosarcoma cells express immune checkpoint ligands such as PD-L1, B7-H3, CD70, and MCAM, which contribute to immune evasion by suppressing T cell and macrophage activity. Additionally, natural killer (NK) cells, which express TIM-3, play a role in targeting osteosarcoma cells, but their activity can be inhibited by tumor-derived signals. Enhancing NK cell function through immunotherapies, such as CAR-NK cells targeting CD70 or MCAM, is another promising therapeutic approach. Collectively, the figure underscores the importance of targeting immune checkpoint pathways (e.g., PD-1/PD-L1, CTLA-4, CD47-SIRPα) and utilizing adoptive cell therapies (e.g., CAR-T and CAR-NK cells) to overcome immune evasion in osteosarcoma and improve treatment outcomes.

Targeting specific antigens in osteosarcoma has emerged as a promising therapeutic strategy. GD2, a disialoganglioside, is highly expressed in pediatric solid tumors, including osteosarcoma (61, 62) (**Figure 1**). Preclinical studies have shown that GD2-targeted CAR-T cells exhibit potent anti- osteosarcoma activity *in vitro* and in mouse models. However, a phase I clinical trial (NCT02107963) evaluating GD2-CAR-T therapy in osteosarcoma patients demonstrated limited efficacy, with only transient tumor regression observed in some patients. The lack of durable responses was attributed to T cell exhaustion and immune escape mechanisms (9). HER2, a human epidermal growth factor receptor, is variably expressed in osteosarcoma, but targeting it remains a viable approach (63, 64). A phase I clinical trial (NCT00902044) testing HER2-CAR-T cells in osteosarcoma patients showed safety but only modest efficacy, with a lack of sustained responses linked to T cell exhaustion and antigen heterogeneity (65). One of the key challenges has been antigen heterogeneity, leading to immune escape and limited response rates. Combination approaches, such as combining HER2-CAR-T cells with checkpoint inhibitors like PD-1 blockade, have shown improved persistence and tumor clearance in preclinical models. The bispecific antibodies (BsAbs) targeting GD2 and HER2 in osteosarcoma demonstrated potent anti-tumor effects both *in vitro* and *in vivo*. Preclinical studies indicate that combining BsAb therapy with anti-PD-L1 blockade enhances T cell activation and tumor clearance. However, clinical validation is still needed to determine the optimal patient selection criteria and therapeutic combinations. T cells armed with these BsAbs showed significant anti-tumor activity, and the combination of BsAbs with anti-PD-L1 antibodies further enhanced the anti-tumor response. These findings support clinical trials investigating GD2 and HER2-targeted T-BsAb therapy in combination with immune checkpoint inhibitors to improve treatment outcomes for osteosarcoma patients (66).

B7-H3 (CD276) is overexpressed in osteosarcoma and plays a significant role in immune evasion (67). The B7-H3-targeting antibody-drug conjugate (ADC) m276-SL-PBD has demonstrated significant antitumor activity in pediatric solid tumor models, including patient-derived (PDX) and cell line-derived xenografts (CDX). In randomized trials, m276-SL-PBD achieved a 92.3% response rate, with 61.5% of models showing a maintained complete response (68). These findings support the clinical development of m276-SL-PBD for high-risk pediatric solid malignancies. Preclinical studies have demonstrated that B7-H3 CAR-T cells induce robust tumor regression in osteosarcoma mouse models. Early-phase clinical trials for B7-H3 CAR-T therapy are ongoing, with promising initial results, highlighting its potential as a target for osteosarcoma immunotherapy. Additionally, recent studies in canine models have shown that B7-H3 CAR-T cells can specifically target and kill B7-H3-expressing canine osteosarcoma cells both *in vitro* and *in vivo*. Furthermore, co-expressing a chemokine receptor (CXCR2) with the B7-H3 CAR construct significantly enhanced the anti-tumor activity of canine CAR-T cells, suggesting a potential strategy to improve CAR-T cell efficacy in osteosarcoma treatment (69).

### 4.2 CAR-NK cell therapy: advantages over CAR-T and potential for clinical translation

CAR-modified NK cells offer several advantages over CAR-T cells in cancer therapy (70). First, they present a lower risk of cytokine release syndrome (CRS) and graft-versus-host disease (GVHD), which are common complications associated with CAR-T cell therapy. Second, NK cells have an innate tumor-killing capacity that is independent of antigen specificity, enabling them to target a wider range of tumor cells. Additionally, CAR-NK cells can function effectively in immunosuppressive tumor microenvironments, making them a promising therapeutic option in resistant cancers. However, a major limitation is the relatively short lifespan of adoptively transferred NK cells, which may reduce long-term efficacy in clinical settings.

#### 4.2.1 Targeting osteosarcoma with CAR-NK Cells

CAR-NK cell therapy has emerged as a promising immunotherapeutic approach for osteosarcoma. Unlike traditional T-cell therapies, CAR-NK cells offer several advantages, including reduced risk of cytokine release syndrome, neurotoxicity, and graft-versus-host disease, thereby enhancing safety profiles (71). Preclinical studies have shown that CAR-NK cells targeting CD70 exhibit enhanced cytotoxicity against osteosarcoma cell lines, but the clinical relevance of CD70 expression in osteosarcoma patients remains to be validated (72, 73). Additionally, anti-melanoma cell adhesion molecule (MCAM) CAR-NK cells have shown significant antitumor activity in osteosarcoma models, suggesting that targeting MCAM could be a viable strategy for OS immunotherapy (74). However, the lack of standardized patient selection criteria and limited data on *in vivo* persistence remain key challenges. Combination approaches are being explored to enhance CAR-NK cell efficacy. For example, the IL-15 agonist NKTR-255 has been shown to prolong NK cell survival and enhance tumor-killing activity in preclinical models (25, 75). Similarly, CD47 blockade using magrolimab (MAG) has been reported to augment CAR-NK cell-mediated cytotoxicity by enhancing macrophage phagocytosis (75). While these approaches are promising, clinical data on the efficacy and durability of CAR-NK cell therapy in osteosarcoma are still limited, highlighting the need for further studies.

### 4.3 Dendritic cell vaccines: enhancing anti-tumor immune priming

DCs are potent APCs that prime T cell responses against tumors. DC-based vaccines aim to enhance anti-tumor immunity by delivering tumor antigens to the immune system. DC-based vaccines represent a promising strategy for osteosarcoma immunotherapy. One approach involves autologous DC vaccines, where patient-derived DCs are loaded with osteosarcoma -associated antigens, such as tumor lysates or synthetic peptides, and reinfused into the patient to stimulate an anti-tumor immune response. A clinical trial using a DC vaccine pulsed with osteosarcoma tumor lysates reported prolonged progression-free survival in a subset of patients, but overall response rates remained low (76). In addition to autologous vaccines, combination strategies have been explored to enhance their effectiveness. Combining DC vaccines with checkpoint inhibitors, such as anti-PD-1 therapy, has shown improved tumor rejection in osteosarcoma models, indicating a synergistic effect (77). Another promising approach is combining DC vaccines with oncolytic viruses. These viruses, engineered to express tumor antigens, can enhance DC-mediated T cell priming, further boosting the anti-tumor immune response (78). Despite these advancements, limited clinical efficacy has been observed, highlighting the need for further optimization of DC vaccine platforms to achieve more consistent and durable therapeutic outcomes (5).

## 5 Combination strategies and emerging approaches

Given the complexity of osteosarcoma’s immune landscape, monotherapy approaches often fail to achieve durable responses. Combination strategies that integrate multiple immunotherapeutic modalities or pair immunotherapy with conventional treatments (e.g., chemotherapy, radiotherapy, and targeted therapy) have demonstrated enhanced efficacy in overcoming immune resistance. Emerging approaches, including oncolytic virotherapy, TLR agonists, and neoantigen-based vaccines, offer new opportunities to boost anti-tumor immunity.

A growing body of evidence suggests that combining immunotherapy with conventional treatment modalities enhance therapeutic efficacy in osteosarcoma by modifying the tumor microenvironment, increasing antigen presentation, and mitigating immunosuppressive mechanisms. Chemotherapy has long been a cornerstone of osteosarcoma treatment, and certain agents such as doxorubicin, cisplatin, and methotrexate have been shown to induce immunogenic cell death (ICD) (79). This process not only leads to tumor cell apoptosis but also enhances the exposure of tumor-associated antigens, thereby stimulating an adaptive immune response. Preclinical studies indicate that checkpoint blockade therapies combined with chemotherapy augment T cell activation and improve response rates in osteosarcoma models (80). However, the immunosuppressive effects of chemotherapy on lymphocytes and antigen-presenting cells remain a challenge, necessitating precise optimization of drug selection, sequencing, and dosing to preserve immune function while maximizing anti-tumor immunity.

Similarly, radiotherapy has emerged as a potent immunomodulator in osteosarcoma by promoting antigen release, increasing tumor immunogenicity, and upregulating immune checkpoint molecule expression such as PD-L1 (81). Ionizing radiation has been shown to enhance T cell infiltration into tumors, rendering osteosarcoma more susceptible to immunotherapy. The concept of the “abscopal effect,” wherein localized radiation induces systemic anti-tumor immune responses, has gained attention as a potential mechanism to enhance immunotherapy efficacy. Despite promising preclinical data, clinical evidence demonstrating the abscopal effect in osteosarcoma remains limited, highlighting the need for well-designed clinical trials. Current pre-clinical exploring the combination of radiotherapy with PD-1/PD-L1 inhibitors, as well as CAR-T cell therapy, to assess their synergistic potential in overcoming immune evasion and improving treatment outcomes in osteosarcoma patients are pending.

Targeted therapies have also been integrated into immunotherapy strategies for osteosarcoma, particularly tyrosine kinase inhibitors (TKIs) such as cabozantinib and sorafenib (82). These agents exhibit immunomodulatory properties by enhancing T cell infiltration, reducing Treg activity, and modulating MDSC populations. Preclinical studies suggest that TKIs increases tumor susceptibility to checkpoint blockade therapy, and clinical trials are currently assessing their combination with PD-1 inhibitors in various solid tumors, including osteosarcoma (83–85). In addition, indoleamine 2,3-dioxygenase (IDO) inhibitors, which target tryptophan metabolism, have been proposed as a strategy to reverse immune suppression in osteosarcoma by limiting the production of immunosuppressive metabolites that impair T cell function (86). However, their clinical success has been inconsistent, suggesting that further mechanistic insights into metabolic immune regulation in osteosarcoma are needed to refine their therapeutic application. Additionally, resistance to immune checkpoint blockade in osteosarcoma is influenced by multiple factors, including tumor mutational burden (TMB), antigen presentation deficiencies, and the immunosuppressive TME. Osteosarcoma is generally characterized by a low TMB, which may contribute to reduced neoantigen presentation and limited T cell recognition. Moreover, defects in antigen processing and presentation, such as the downregulation of MHC-I molecules, can impair immune detection and contribute to therapeutic resistance. The TME further exacerbates resistance by fostering an immunosuppressive milieu dominated by MDSCs, Tregs, and TAMs, all of which inhibit effective anti-tumor immunity. Addressing these resistance mechanisms through combination approaches, such as incorporating epigenetic modulators, metabolic reprogramming strategies, and novel immune checkpoint targets, is essential for enhancing response rates and improving clinical outcomes in osteosarcoma.

Despite the promise of combination strategies, several challenges remain in optimizing their clinical application. The timing, dosing, and sequencing of these therapies must be carefully calibrated to balance tumor cytotoxicity with immune stimulation while minimizing toxicity. Furthermore, osteosarcoma exhibits significant molecular heterogeneity, necessitating a personalized approach that incorporates molecular profiling and immune biomarkers to tailor treatment regimens for individual patients (87, 88). A deeper understanding of osteosarcoma’s immune microenvironment heterogeneity is crucial to identifying patient subgroups that may benefit most from specific combination strategies.

Advances in systems biology, machine learning, and high-throughput screening may aid in identifying optimal combination strategies that maximize therapeutic efficacy while mitigating adverse effects.

## 6 Conclusion

Immunotherapy has emerged as a promising strategy for osteosarcoma, a malignancy with historically limited treatment options and poor outcomes in relapsed or metastatic cases (5). Over the past decade, significant advances have been made in understanding the immunosuppressive tumor microenvironment, leading to the development of various immune-based interventions. Checkpoint blockade therapies, cellular therapies such as CAR-T and CAR-NK cells, and combination strategies integrating oncolytic virotherapy, microbiome modulation, Toll-like receptor agonists, and neoantigen-based vaccines have shown potential in preclinical and early-phase clinical studies. However, despite these advances, the clinical efficacy of immunotherapy in osteosarcoma remains inconsistent due to tumor heterogeneity, immune evasion mechanisms, and insufficient immune infiltration.

To translate these promising findings into meaningful clinical benefits, further translational research and well-designed clinical trials are essential. The identification of predictive biomarkers will be crucial for patient stratification, ensuring that the most suitable patients receive immunotherapy-based interventions. Currently, a major limitation is the lack of validated biomarkers for predicting responses to immunotherapy, necessitating further exploration of molecular and immune profiling strategies. Additionally, the development of next-generation cellular therapies, including multi-targeted CAR-T cells, armored CAR-NK cells, and gene-edited immune cells, holds great promise for overcoming resistance and improving treatment durability.

Looking ahead, a multidisciplinary approach that combines immunotherapy with conventional treatments such as chemotherapy, radiotherapy, and targeted therapy may pave the way for more effective, personalized treatment regimens. The integration of AI-driven precision medicine and liquid biopsy-based monitoring may refine patient selection and treatment adaptation, increasing therapeutic success rates. As research continues to advance, overcoming key barriers—such as immune evasion, inadequate T cell infiltration, and therapy resistance—will be essential for improving clinical outcomes. Further investigation into emerging strategies, including the use of oncolytic viruses to enhance tumor immunogenicity, microbiome-based interventions to modulate systemic immunity, and neoantigen-based vaccines for personalized immunotherapy, may provide novel avenues to enhance treatment efficacy. Cellular and immune-based therapies are expected to play an increasingly central role in improving osteosarcoma outcomes, offering new hope for patients with this aggressive malignancy.
